# Phase Equilibrium, Morphology, and Physico-Mechanics in Epoxy–Thermoplastic Mixtures with Upper and Lower Critical Solution Temperatures

**DOI:** 10.3390/polym13010035

**Published:** 2020-12-24

**Authors:** Alexey V. Shapagin, Nikita Yu. Budylin, Anatoly E. Chalykh, Vitaliy I. Solodilov, Roman A. Korokhin, Arkadiy A. Poteryaev

**Affiliations:** 1 Frumkin Institute of Physical Chemistry and Electrochemistry Russian Academy of Sciences (IPCE RAS), 31, bld.4 Leninsky Prospect, 119071 Moscow, Russia; budylin_nikita@mail.ru (N.Y.B.); chalykh@mail.ru (A.E.C.); apoteryaev@gmail.com (A.A.P.); 2 N.N. Semenov Federal Research Center for Chemical Physics Russian Academy of Sciences (FRCCP RAS), 4, Kosygina str., 119991 Moscow, Russia; vital-yo@yandex.ru (V.I.S.); korohinra@gmail.com (R.A.K.)

**Keywords:** polymer, phase equilibrium, morphology, phase structure, epoxy, polysulfone, polyethersulfone, curing, elastic modulus, tensile strength, crack resistance

## Abstract

The mutual solubility of epoxy oligomer with polysulfone (PSU) and polyethersulfone (PES) was studied by optical interferometry. Additionally, phase diagrams (PDs) were plotted and their evolution during the curing process was shown. The phase structures of modified hardened systems, as well as their tensile strengths, elastic moduli, and crack resistance, have been studied by scanning electron microscopy and physico-mechanical techniques. The effect of initial components’ mutual solubility on the phase structure and, subsequently, on the physico-mechanical properties of the composite material is shown. Differences in the structure and properties of the cured modified compositions depending on the type of PD (with Upper Critical Solution Temperature (UCST) for PSU and Lower Critical Solution Temperature (LCST) for PES) of the initial components are shown.

## 1. Introduction

The development of the aerospace, automotive, and construction industries leads to the increase in the requirements for polymer binders for composite materials. In order to improve physico-mechanical and thermophysical properties, epoxy binders are modified with thermoplastic rigid-chain polymers (TPs) [1,2,3,4,5,6,7,8,9,10,11,12,13]. Currently, a large number of publications are devoted to the structure and properties of cured compounds with given compositions [2,3,4,5,6,7,8,9,10,11,12]. However, very few studies are devoted to changes in the mutual solubility of the components of the composition during the curing reaction. Previously, we have shown that parameters of the critical point of a phase diagram (PD) and its distance from the curing isotherm determine the type of the forming phase structure and sizes of the phases [14,15]. Thus, the properties of heterogeneous systems are dependent on the mutual solubility and translational mobility of the components and kinetics of the chemical reaction, which affect the formation of the phase structure of the composite material and their properties [8,9,10,11,12,15,16,17,18,19,20,21].

According to [7,12,15], mixtures of an epoxy oligomer (EO) with rigid-chain thermoplastics such as polysulfone and polyethersulfone (which have similar molecular structures) are characterized by PD of amorphous separation with upper and lower critical solution temperatures, respectively.

The current paper is devoted to studying the effect of the solubility of the components before and during curing reactions of epoxy–TP systems on the structure of the cured composition as well as establishing a correlation between the main physical and mechanical properties and the phase structures of cured epoxy systems modified with polysulfone (PSU) and polyethersulfone (PES). The results of the study are of great importance for technologists when selecting composition of the binder to obtain the required structure of the cured matrix, which determines the maximum physical and mechanical characteristics of the products used in aerospace, automotive, and construction industries.

## 2. Experimental

The epoxy oligomer (ED-20, Armplast, Moscow, Russia) with M_w_ = 0.35 × 10^3^ g/mol, T_g_ = −18 °C, PSU (PSK-1, Petrov Research Institute of Plastics, Moscow, Russia) with M_w_ = 35 × 10^3^ g/mol, T_g_ = 180 °C and PES (BASF Corporation, Wyandotte, MI, USA) with M_w_ = 34 × 10^3^ g/mol, T_g_ = 240 °C were chosen as objects of the study. Triethanolamine titanate (TEAT) was used as a hardener (T_curing_ = 160 °C, stoichiometric ratio 10 wt.%) [22].

Partially cured EO with a nonstoichiometric content of TEAT (adduct) was used to study the evolution of PD. Adduct curing degree (α) was calculated by the ratio of thermal effects of adducts and fully cured oligomers with the stoichiometric content of TEAT, recorded by the differential scanning calorimetry (DSC) method (Netzsch DSC 204 F1, Selb, Germany). Each sample was measured in inert atmosphere from 20 °C until 260 °C with 5K per minute heating rate.

The study of solubility and interdiffusion in binary (PSU–EO, PES–EO) and three-component systems—TPs–adducts—was carried out using method of optical interferometry on an ODA-2 IPCE diffusiometer (IPCE, Moscow, Russia) [23]. A helium–neon laser (λ = 632.8 nm) was used as a light source.

The method is based on the principle of in situ registration of optical density distribution in the area of conjugation of components and recording its change in time under the isobaric–isothermal conditions of the process [24]. The measurement method consisted in fixation of a TP sample of 5 mm × 5 mm in size and about 150 μm thick (obtained by pressing) between the diffusion cell glasses, the inner surfaces of which are covered with a layer of translucent metal (Ni-Cr alloy) with a high reflection index. A small wedge angle of 2° was established between the glasses. After assembly, the cell was thermostated at a set temperature for at least 30 min. Then, the space between the glasses was filled with EO or adduct.

All measurements were carried out in the temperature range from 20 to 260 °C. The experiments was carried out in the heating–cooling mode with a step of 5 °C and thermostated at each stage for at least 30 min.

Methods of processing of the interferograms, interdiffusion zones, and phase diagrams construction did not differ from those described earlier [25,26].

The phase structure and physico-mechanical properties of epoxy–TP systems PSU–adduct and PES–adduct with TP content from 5 to 20 wt.% were studied using standard mixed samples, hardened for 8 h at 160 °C. The choice of such a concentration range is related to the manufacturability of epoxy composite materials. Epoxy binders with contents of rigid-chain thermoplastics of more than 20 wt.% have high viscosity and cannot be used to impregnate fibers and fabrics.

The crack morphology and elemental composition of phases were examined on a scanning electron microscope (Philips SEM-500, Eindhoven, The Netherlands) with X-ray energy spectrometer (Kevex-Ray, Burlingame, CA, USA).

Phase structure was elicited by plasma etching of a low-frequency oxygen discharge with the vacuum universal station (Edwards Coating System E306A, UK).

Physico-mechanical properties of the samples—elastic modulus (E), tensile strength (σ) and crack resistance (G_IR_)—were assessed using the universal testing machine (Zwick/Roell Z010, Ulm, Germany) under tension at a constant rate of 1 mm/min.

**Figure 1:**
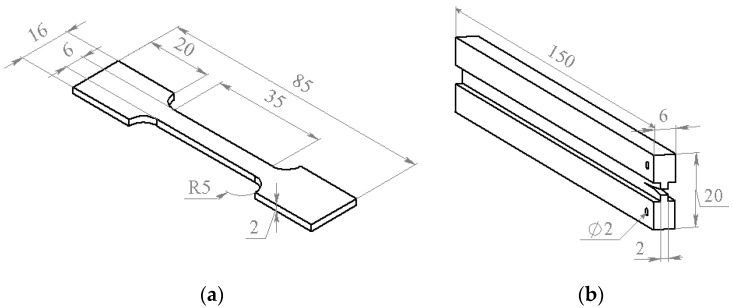
The specimens used for the tensile strength (**a**) and the crack resistance (**b**) measurements.

For elastic modulus (E) and tensile strength measurements, the specimens shown in Figure 1a were used. In the tests, applied force (P) and strain (ε) were registered. The strain was measured with the MultiXtens extensometer. Tensile strength and elastic modulus were calculated from obtained stress–strain curves.

The samples shown in Figure 1b were used for the crack resistance measurements. Thickness of the groove used for directing the crack did not exceed 2 mm. Initial notch for crack initiation with the 0.1 mm thickness was made by the razor blade and situated 8–10 mm away from the through holes for the sample fixation in the universal testing machine (UMT) grips. Under loading, the force increased until a certain critical value when the crack started to propagate. After the crack propagation stopped, the sample was unloaded and the loading cycles were repeated until the final cracking of the sample. The technique used for the crack resistance measurements was described in [27].

## 3. Results and Discussion

### 3.1. Phase Equillibrium

**Figure 2:**
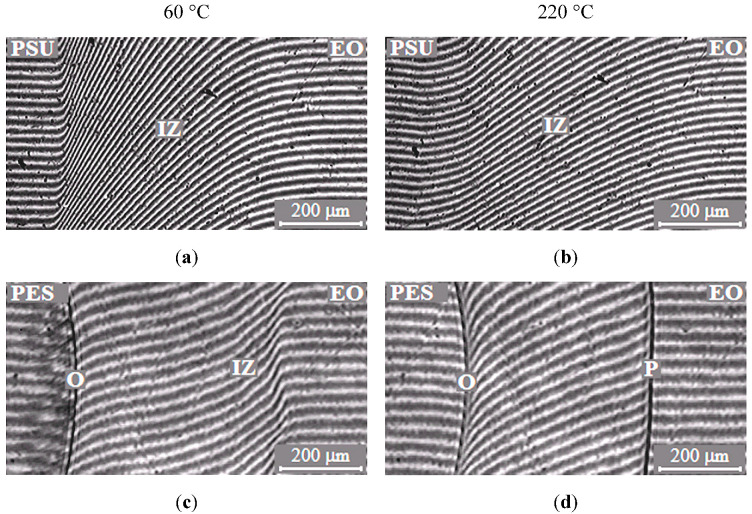
Typical interferograms of interdiffusion zones of the polysulfone (PSU)–epoxy oligomer (EO) systems (**a**,**b**) and polyethersulfone (PES)–EO (**c**,**d**) at 60 (**a**,**c**) and 220 °C (**b**,**d**). O is the optical boundary, IZ is the interdiffusion zone and P is the phase boundary.

The typical interferograms of interdiffusion zones for systems PSU–EO and PES–EO are presented in Figure 2. It was established that EO mixtures with PSU are fully compatible in the temperature range 20–260 °C, since the refractive index (n) continuously changes from n_TP_ to n_oligomer_ (Figure 2a,b).

In contrast to the mixtures of EO with PSU, the EO systems with PES have phase boundaries in the area of dilute solutions, which indicates partial compatibility of PES–EO. It was established that, at low temperatures (below 90 °C), mixture of EO with PES is characterized by a complete solubility (Figure 2c) and, at higher temperatures (above 90 °C), there is a partial compatibility (Figure 2d). Thus, the PES–EO system belongs to the class of systems with amorphous phase separation with Lower Critical Solution Temperature (LCST).

**Figure 3:**
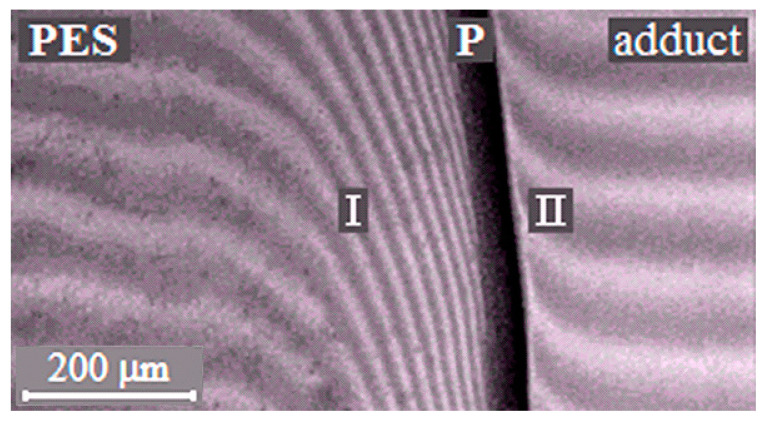
Interferograms of interdiffusion zones of the PSU–adduct system (α = 0.22, T = 220 °C). P is the phase boundary, I is the diffusion zone of the adduct into the thermoplastic rigid-chain polymer (TP) and II is the diffusion zone of the TP into the adduct.

**Figure 4:**
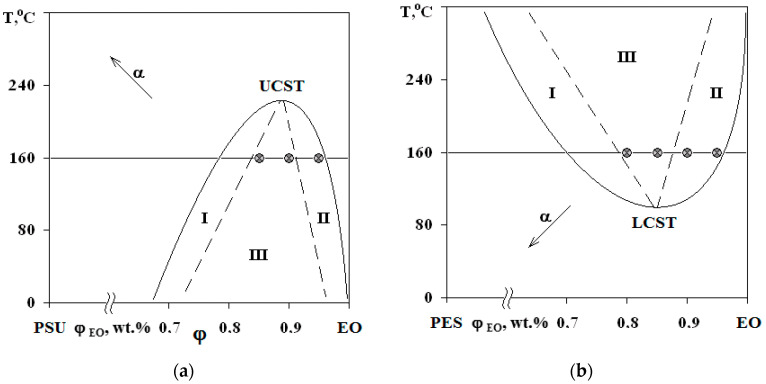
PDs of EO mixtures with PSU at α = 0.25 (**a**) and with PES at α = 0 (**b**). Regions I and II—structures of the “matrix-inclusion” type (sea-island); III—phase reversal region. The compositions of studied systems are marked on the isotherm at 160 °C.

The phase boundary (Figure 3), separating the dissolution areas of oligomers in the TP (I) and the TP in the partially hardened EO (II), appears in the interdiffusion zone of the PSU–adduct system, compared to original linear systems. The systems are characterized by a PD of amorphous phase separation with Upper Critical Solution Temperature (UCST) (Figure 4a). It was established that after the system has overcome the gel-point, the phase boundary presents under all temperature and temporal conditions to the point of polymer decomposition. Equilibrium state of the system is confirmed in the reversed heating–cooling mode.

Interference patterns were analyzed using the standard technique [25]. The compositions of coexisting phases were determined and PDs were constructed at different stages of EO crosslinking. Phase inversion areas on the PD are indicated by the dashed lines on the basis of structural and morphological studies of cured compositions. Arrows (Figure 4) indicate the shift direction of critical point and binodal dome, which occurs due to the formation of spatial network of chemical bonds. It was established that an increase in the network density leads to a decrease in the solubility of components, an increase in UCST for PSU–EO systems and a decrease in the LCST for PES–EO systems. Compositions of coexisting phases change and the diffusion coefficient decreases due to the chemical reactions of crosslinking and network formation, which leads to the effect of concentration supersaturation, creating conditions for secondary phase structure formation.

### 3.2. Phase Structure

The phase structure of cured epoxy–TP compositions with UCST and LCST was studied on the mixtures with TPs at a concentration range of 5–20 wt.%. The figurative points (FPs) of the studied mixtures are plotted on a PD (Figure 4).

**Figure 5:**
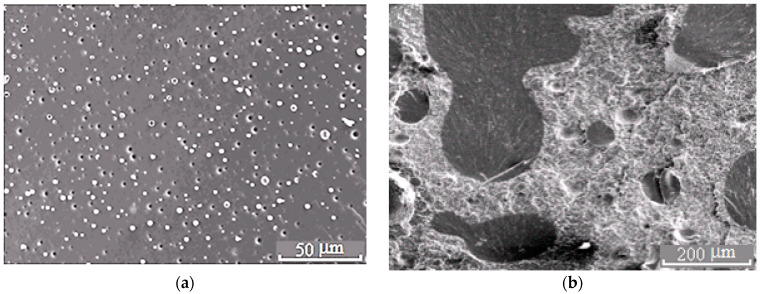
Morphology of the PSU–EO mixture at φ_PSU_ 5 (**a**) and 15 wt.% (**b**).

**Figure 6:**
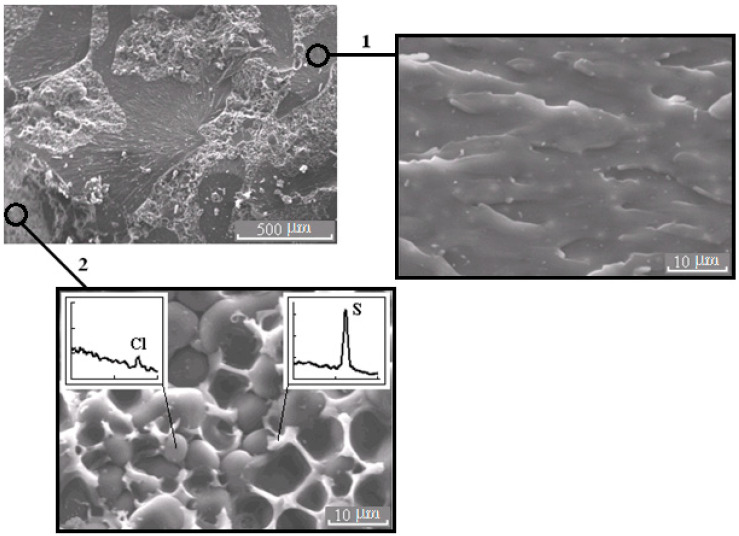
Morphology of the mixture PSU–EO at φ_PSU_ 10 wt.% with bicontinuous type of structure; 1 and 2 are regions enriched in epoxy polymer and TP, respectively. On the inserts, fragments of the X-ray spectra of microphases are represented.

**Figure 7:**
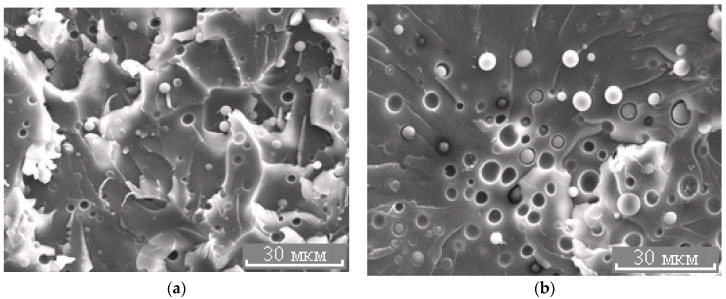
Morphology of the PES–EO mixture at φ_PES_ 5 (**a**) and 10 wt.% (**b**).

**Figure 8:**
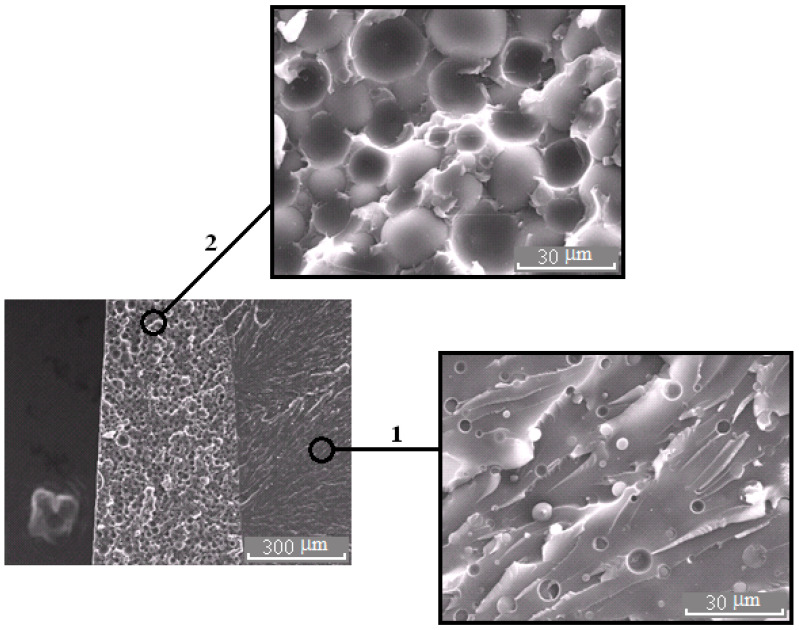
Morphology of the PES–EO mixture at φ_PES_ 15 wt.%.

**Figure 9:**
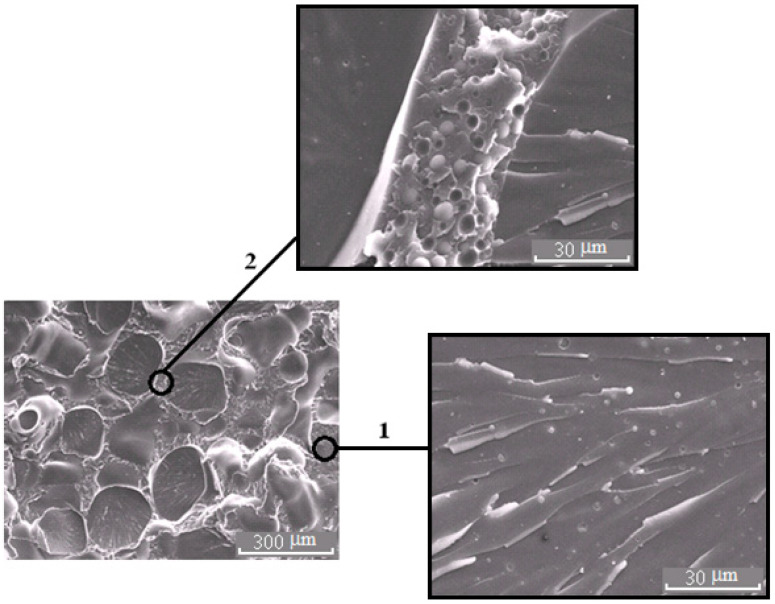
Morphology of the PES–EO mixture at φ_PES_ 20 wt.%.

The SEM micrographs of phase structures of cured systems with UCST and LCST are represented in Figure 5, Figure 6, Figure 7, Figure 8 and Figure 9. All compositions have heterogeneous structures. Their specifics are manifested in various types of phase structure organizations, particle size distributions and presence of secondary phase transition particles.

Phase structure formation in systems with PSU and PES occurs at 160 °C. However, in systems with LCST, the phase separation begins at the stage of temperature increase, since the path of the FP movement crosses the binodal curve at the point of 90 °C.

**Figure 10:**
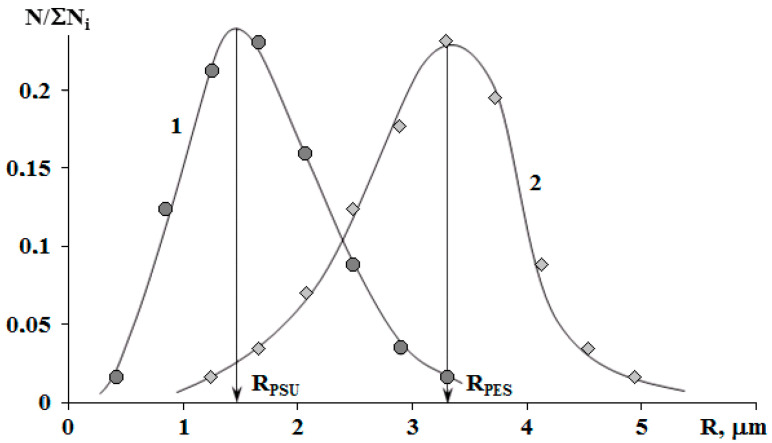
Size distribution of phases in the cured mixtures PSU–EO (1) and PES–EO (2) with 5 wt.% of TP. R_PSU_ and R_PES_ are the average sizes of heterogeneous particles in the PSU–EO and PES–EO systems, respectively.

The binodal dome intersects the FPs of systems with PSU at a conversion degree of 0.25. Thus, considering nearly equal molecular weights of TPs, phase separation in the system with PSU occurs at lower interdiffusion coefficients than in systems with PES, which directly affects the size of the dispersed phase. It was established that in systems with 5 wt.% TP, the phase distribution is unimodal. The average size of dispersion particles in the system with PES is 3.3 μm, and with PSU it is 1.5 μm (Figure 10). Thereby, the sizes of dispersed structures are mostly determined by the conversion rate, at which the formation of heterogeneous structures begins when the FP enters the labile area of PD.

It is important that the formation of bicontinuous “salami” type structures occurs with different molecular mobilities of the system components, in the case of systems with PSU at α = 0.25, and, for PES, when the temperature rises from 90 to 160 °C (before the start of the curing reaction). Therefore, the dispersed structures formed at secondary phase separation are larger in the case of systems with PES. Oligomer-rich phases in systems with PES have a grain size approximately 20 μm (Figure 8), and in the systems with PSU grain size does not exceed 10 μm (Figure 6) in the 2 region.

**Figure 11:**
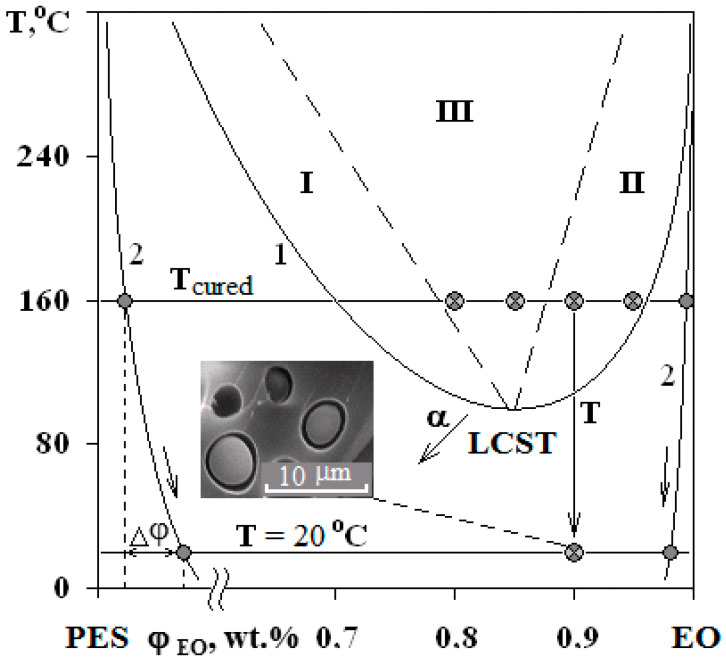
Evolution of the PD of the PES–EO system during the curing reaction. The arrow α indicates the direction of the critical temperature shift with increasing α. Other arrows indicate the change in the composition of the phases with decreasing temperature of the cured system. Δφ is the composition shift of the dispersed phase in the cured mixture at room temperature. The micrograph shows the phase structure of the mixture at 10 wt.% of PES.

The micrograph (Figure 7b and Figure 11) shows that the dispersed phase is smaller in size compared to the well region. In our opinion, this effect is associated with an interfacial adhesion contact failure, which is caused by shrinkage stresses and change in dispersed phase composition of cured system cooled from curing temperature to the room temperature at Δφ (left binodal 2 in Figure 11) while the matrix composition changes slightly (right binodal 2 in Figure 11). It should be noted that the PSU–EO system with UCST does not have the same phenomenon due to an increase in TP concentration in the dispersed phase as a result of cooling.

Thus, the type of phase structure formed during curing of the mixture is influenced by the critical concentration—the intersection point of the binodal curve with curing isotherm. If composition of the mixture under the curing equals to the critical concentration, complex bicontinuous structures (salami type) will be formed in the system. If critical concentration shifts by more than 5 wt.% in either direction, the morphology of a cured composition will correspond to a matrix-dispersion type of structure (sea-island) with a dispersed phase enriched with one of the components. The critical concentration value can be adjusted by varying the molecular weights of the initial system components, according to the equation $\varphi_{kp2}=\frac{\sqrt{r_{1}}}{\sqrt{r_{1}}+\sqrt{r_{2}}}$ following from the theory of Flory–Huggins–Scott of polymer solutions for amorphous systems [28], where r_1_ and r_2_ are the degrees of polymerization of components 1 and 2.

It is important that the effective molecular weight of the oligomer continuously changes at the curing process, which leads to expansion of heterogeneous region of PD and, as a result, the continuous change in the composition of coexisting phases.

Therefore, due to the low diffusion coefficients in the final stages of the curing reaction, concentration supersaturation appears. This provokes secondary phase transformations and, as a result, nanoscale structure formation. It is the molecular mobility of system components during the curing reaction that determines the size of the final phase structures.

### 3.3. Physico-Mechanical Properties

As it was shown above, regardless of the type of original PD, characterized by UCST or LCST, the main factors affecting the final phase structure of composition are the molecular weights of the mixture components and the differences between the critical parameters (concentration, temperature) and the curable mixture composition and the curing temperature, respectively. Their variation allows the obtention of compositions with the necessary structural parameters that determine the set of physico-mechanical and other operational characteristics of the sample.

**Figure 12:**
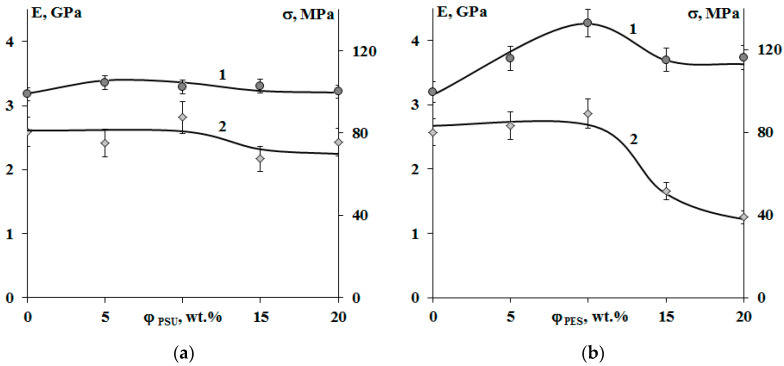
Concentration dependences of the elastic modulus (1) and tensile strength (2) of cured epoxy–TP compositions modified with PSU (**a**) and PES (**b**). Temperature −20 °C.

Figure 12 shows that modification of systems with PSU induces changes in the elastic modulus and tensile strength within the error range, when there is a decrease in tensile strength by two times in the area of phase inversion (φ_PES_ 15 and 20 wt.%) for systems with PES. An increase in the size of PES-rich dispersed phase leads to a 25% increase in the modulus of elasticity. This effect is not observed for systems with PSU. Growth of elastic modulus of compositions modified with PES at concentrations up to 10 wt.% is due to a formation of heterogeneous dispersion of larger particles in the structure. It is important that, at low modifier concentrations, the dispersed phase is enriched with TP with a higher T_g_ (240 °C), and hence higher elastic modulus, than the cured EO, whose T_g_ is comparable to the curing temperature (160 °C). The tensile strength loss occurring in EO–PES systems (φ_PES_ 15 and 20 wt.%) with “salami” type structures is associated with a phase composition change (Δφ) resulting from the composition being cooled to room temperature (Figure 11).

**Figure 13:**
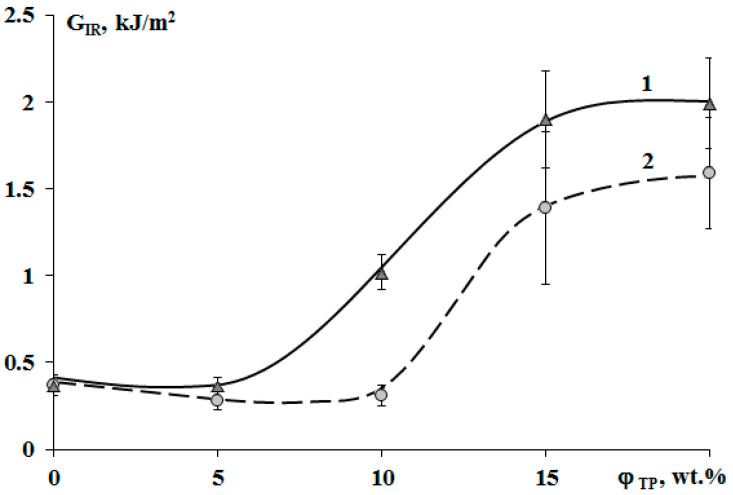
Concentration dependences of crack resistance (G_IR_) of cured epoxy–TP compositions modified with PSU (1) and PES (2).

The crack resistance is improved for “salami” type structures. It was found that addition of 10 wt.% of PSU and 15% of PES increased the crack resistance (Figure 13). This effect can be explained by appearance of a continuous phase enriched with a thermoplastic component. For TP concentration of 20 wt.%, crack resistance of the PES–EO composition was 20% lower than of the PSU–EO one. This is due to the lower values of PSU elastic modulus compared to PES one, which facilitates a decrease in the crack growth energy.

## 4. Conclusions

A number of important observations from the present work shed more light on the processes occurring during the curing of modified binders. The correlation between the phase equilibria, structure and physico-mechanical properties of epoxy systems modified by PSF and PES was determined.

Information on the phase state of the systems under study in the entire concentration and wide temperature (20–260 °C) ranges has been obtained. It is shown that mixtures of EO with PSF and PES are characterized by PD with upper and lower critical solution temperatures, respectively. The influence of the thermal prehistory on the phase structure regulation depending on the position of the figurative point on the PD was traced (concentrations of components).

The formation of bicontinuous “salami” phase structures leads to increases in the crack resistance. It was determined that in order to obtain this type of structure in the cured TP–EO system, the TP concentration should be equal to the critical concentration, when the binodal intersects the curing isotherm on the PD. It was found that in epoxy systems modified with PES (PD with LCST) and the matrix-dispersion type of structure with interfacial adhesion, contact failure occurs.

The obtained information is of great importance in the engineering of products from composite materials based on epoxy binders modified PSF and PES and allows one to predict the matrix structure and, as a consequence, the physical and mechanical properties when choosing the binder composition.

## Data Availability

The data presented in this study are available on request from the corresponding author.

## References

[1] Farooq U., Teuwen J., Dransfeld C. (2020). Toughening of Epoxy Systems with Interpenetrating Polymer Network (IPN): A Review. Polymers.

[2] Rosetti Y., Alcouffe P., Pascault J.P., Gerard J.F., Lortie F. (2018). Polyether Sulfone-Based Epoxy Toughening: From Micro- to Nano-Phase Separation via PES End-Chain Modification and Process Engineering. Materials.

[3] Korokhin R.A., Solodilov V.I., Zvereva U.G., Solomatin D.V., Gorbatkina Y.A., Shapagin A.V., Lebedeva O.V., Bamborin M.Y. (2020). Epoxy polymers modified with polyetherimide. Part II: Physicomechanical properties of modified epoxy oligomers and carbon fiber reinforced plastics based on them. Polym. Bull..

[4] Zhang Y.W., Shen Y.C., Shi K.X., Wang T.W., Harkin-Jones E. (2018). Constructing a filler network for thermal conductivity enhancement in epoxy composites via reaction-induced phase separation. Compos. Part A Appl. Sci. Manuf..

[5] Kishi H., Tanaka S., Nakashima Y., Saruwatari T. (2016). Self-assembled three-dimensional structure of epoxy/polyethersulphone/silver adhesives with electrical conductivity. Polymer.

[6] Chalykh A.E., Gerasimov V.K., Bukhteev A.E., Shapagin A.V., Kudryakova G.K., Brantseva T.V., Gorbatkina Y.A., Kerber M.L. (2003). Compatibility and phase structure evolution in polysulfone-curable epoxy oligomer blends. Polym. Sci. Ser. A.

[7] Chen Z.G., Luo J., Huang Z., Cai C.Q., Tusiime R., Li Z.Y., Wang H.X., Cheng C., Liu Y., Sun Z.Y. (2020). Synergistic toughen epoxy resin by incorporation of polyetherimide and amino groups grafted MWCNTs. Compos. Commun..

[8] Sun Z.Y., Xu L., Chen Z.G., Wang Y.H., Tusiime R., Cheng C., Zhou S., Liu Y., Yu M.H., Zhang H. (2019). Enhancing the Mechanical and Thermal Properties of Epoxy Resin via Blending with Thermoplastic Polysulfone. Polymers.

[9] Korokhin R.A., Solodilov V.I., Gorbatkina Y.A., Shapagin A.V. (2015). Rheological and physicomechenical properties of epoxy-polyetherimide compositions. Mech. Compos. Mater..

[10] Jiang M.Q., Liu Y., Cheng C., Zhou J.L., Liu B.H., Yu M.H., Zhang H. (2018). Enhanced mechanical and thermal properties of monocomponent high performance epoxy resin by blending with hydroxyl terminated polyethersulfone. Polym. Test..

[11] Solodilov V.I., Korokhin R.A., Gorbatkina Y.A., Kuperman A.M. (2015). Comparison of fracture energies of epoxypolysulfone matrices and unidirectional composites based on them. Mech. Compos. Mater..

[12] Mimura K., Ito H., Fujioka H. (2000). Improvement of thermal and mechanical properties by control of morphologies in PES-modified epoxy resins. Polymer.

[13] Surendran A., Joy J., Parameswaranpillai J., Anas S., Thomas S. (2020). An overview of viscoelastic phase separation in epoxy based blends. Soft Matter.

[14] Shapagin A.V., Budylin N.Y., Chalykh A.E. (2018). Regulation of a phase structure at the interface in epoxy-polysulfone systems. Russ. Chem. Bull..

[15] Chalykh A.E., Shapagin A.V. (2008). Phase equilibrium and phase structure of cured polymer nanocompositions. Modern Problems of Physical Chemistry of Nanomaterials.

[16] Won J.S., Lee J.E., Park J.K., Lee M.Y., Kang S.H., Lee S.G. (2019). Cure Behavior and Toughness Properties of Polyethersulfone/Multifunctional Epoxy Resin Blends. Polym. Korea.

[17] Brantseva T.V., Solodilov V.I., Antonov S.V., Gorbunova I.Y., Korohin R.A., Shapagin A.V., Smirnova N.M. (2016). Epoxy modification with poly(vinyl acetate) and poly(vinyl butyral). I. Structure, thermal, and mechanical characteristics. J. Appl. Polym. Sci..

[18] Chistyakov E.M., Terekhov I.V., Shapagin A.V., Filatov S.N., Chuev V.P. (2019). Curing of Epoxy Resin DER-331 by Hexakis(4-acetamidophenoxy)cyclotriphosphazene and Properties of the Prepared Composition. Polymers.

[19] Solodilov V.I., Gorbatkina Y.A., Korokhin R.A., Kuperman A.M. (2018). Properties of Filament-Wound Organoplastics Based on Epoxy Polysulfone Matrices and Armos and Rusar Aramid Fibers. Polym. Sci. Ser. D.

[20] Foraboschi P. (2015). Analytical model to predict the lifetime of concrete members externally reinforced with FRP. Theor. Appl. Fract. Mech..

[21] Foraboschi P. (2016). Effectiveness of novel methods to increase the FRP-masonry bond capacity. Compos. Part B Eng..

[22] Ellis B. (1993). Chemistry and Technology of Epoxy Resins.

[23] Chalykh A.E., Zagaitov A.L., Korotchrnko D.P. (1996). Optical Diffusiometer.

[24] Malkin A., Ascadsky A., Kovriga V., Chalykh A.E. (1983). Experimental Methods of Polymer Physics.

[25] Chalykh A.E., Gerasimov V.K., Mikhailov Y.M. (1998). Phase State Diagrams of Polymer Systems.

[26] Nikulova U.V., Chalykh A.E. (2020). Phase Equilibrium and Interdiffusion in Poly(Vinyl Methyl Ether)-Water System. Polymers.

[27] Babaevskii P.G. (1980). Tutorial in Polymer Materials Science.

[28] Paul D.R., Bucknall C.B. (2000). Polymer Blends.

